# Supplementary material for: Basic school pupils’ food purchases during mid-morning break in urban Ghanaian schools
Source: PLoS One. 2020 Sep 1;15(9):e0238308. doi: 10.1371/journal.pone.0238308 (PMC7462272; doi:10.1371/journal.pone.0238308)
Supplement: S1 File — (DOCX) [file pone.0238308.s001.docx]

QUESTIONNAIRE TO DOCUMENT FOODS FREQUENTLY PURCHASED BY PUPILS

**SCHOOL OF PUBLIC HEALTH, UNIVERSITY OF GHANA**

**DEPARTMENT OF POPULATION, FAMILY & REPRODUCTIVE HEALTH**

**A STUDY TO ASSESS THE DETERMINANTS OF OBESITY AMONG BASIC SCHOOL PUPILS IN ACRRA**

1. Date of interview (dd/mm/yy): DATE __ __/__ __/__ __
2. Name of school ……………………………………. School ID SCHID
3. Interception number:
4. Name of Respondent: …………………………………….
5. Age of Respondent (completed years):
6. Sex of respondent: 1. Male 2. Female
7. School Type: 1. Public 2. Private

**Codes for type of food**:

1. Soft/fizzy drink 2. Sweetened drink 3. Pastry 4. Packaged snacks (Biscuit) 5. Local snack(dry)

6. Fried foods 7. Confectionaries (Candy bar/ lollipop/sweets, etc) 8. Milk/ cocoa drink 9. Complete meal 10. Ice cream 11. Fruits 12. Other (specify)………………………………..

| Item name | Type | Amount (g) | Unit Cost (Ghc) | Place of purchase  Inside sch; 2= outside school |
| --- | --- | --- | --- | --- |
| 1. |  |  |  |  |
| 2. |  |  |  |  |
| 3. |  |  |  |  |
| 4. |  |  |  |  |
| 5. |  |  |  |  |
| 6. |  |  |  |  |
| 7. |  |  |  |  |
| 8. |  |  |  |  |
| 9. |  |  |  |  |
| 10. |  |  |  |  |
