# Supplementary material for: Basic school pupils’ food purchases during mid-morning break in urban Ghanaian schools
Source: PLoS One. 2020 Sep 1;15(9):e0238308. doi: 10.1371/journal.pone.0238308 (PMC7462272; doi:10.1371/journal.pone.0238308)
Supplement: S1 Table — (DOCX) [file pone.0238308.s002.docx]

S2 Table. Energy Density of Foods Frequently Purchased by Pupils at School

| food item | Energy density Kcal/g | typical portion size (g) | Cost (GhS) | Typical Calories (Kcal) |
| --- | --- | --- | --- | --- |
| **Sweetened beverages & candies** | |  |  |  |
| Asana (malted maize drink) +milk | 0.86 | 224 | 0.3 | 192.64 |
| juicee | 0.55 | 212 | 0.6 | 116.6 |
| cocoa drink | 0.1 | 161 | 0.3 | 16.17 |
| skimmed milk ice cream (local) | 0.36 | 118 | 0.2 | 42.598 |
| sobolo/ hibiscus iced tea | 0.37 | 80 | 0.2 | 29.6 |
| new star drink/ u-fresh drink | 0.42 | 80 | 0.1 | 33.68 |
| ice cream (fanice) | 0.81 | 94 | 0.7 | 76.14 |
| soy milk | 0.33 | 66 | 0.1 | 21.78 |
| caramel candy | 4.89 | 7 | 0.1 | 34.23 |
| milk candy | 4.19 | 18 | 0.2 | 75.42 |
| mint toffee | 3.45 | 5 | 0.05 | 17.25 |
| **Snacks** |  |  |  |  |
| Cookies | 4.46 | 12 | 0.3 | 53.52 |
| chips (pastry) | 4.96 | 56 | 0.4 | 277.76 |
| Crackers | 4.76 | 16 | 0.1 | 76.16 |
| digestive biscuit (lola) | 3.19 | 15 | 0.05 | 47.85 |
| cake dougnut | 4.22 | 43 | 0.2 | 181.46 |
| jack n jill biscuit | 0.75 | 40 | 0.7 | 30 |
| Jack n jill fun o biscuit | 1.2 | 57 | 0.6 | 68.4 |
| Biscuit | 3.95 | 27 | 0.1 | 106.65 |
| Bourbon biscuit | 4.46 | 41 |  | 182.86 |
| milk shortcake biscuit | 3.46 | 92 | 0.7 | 318.32 |
| spring roll | 1.78 | 37 | 0.2 | 65.86 |
| Bofrot | 2.76 | 61 | 0.2 | 168.36 |
| ‘Atsomo’(fried flour) | 3.25 | 55 | 0.5 | 178.75 |
| ‘Koose’ (deep fried cowpea paste) | 2.77 | 22 | 0.2 | 60.94 |
| meat pie | 2.89 | 49 | 0.2 | 141.61 |
| Bread | 2.75 | 51 | 0.2 | 140.25 |
| **Complete meals** |  |  |  |  |
| Banku (cooked fermented maize+cassava dough) | 1.08 | 179 | 0.3 | 193.32 |
| banku (30p) + okro soup | 0.86 | 365 | 0.3 | 315 |
| beans and gari + palm oil | 2.28 | 336 | 0.3 | 766.08 |
| tom brown porridge | 0.47 | 182 | 0.3 | 85.722 |
| Waakye | 1.3 | 208 | 0.5 | 270.4 |
| wheat porridge | 0.54 | 256 | 0.6 | 138.5 |
| Ga kenkey (kenkey is cooked fermented maize dough) | 1.24 | 119 | 0.3 | 147.56 |
| groundnut soup (any 20p fish/food purchased) | 1.17 | 408 | 0.2 | 477.36 |
| hausa koko (millet meal porridge)+ sugar | 0.37 | 431 | 0.4 | 157.32 |
| ice kenkey | 0.49 | 247 | 0.4 | 120.78 |
| indomie/ noodles | 1.24 | 148 | 0.6 | 183.52 |
| jollof rice | 1.52 | 356 | 0.5 | 541.12 |
| rice porridge + sugar+milk | 0.58 | 476 | 0.5 | 276.08 |
| riceball (20p) + groundnut soup | 1.27 | 153 | 0.2 | 194.31 |
| ‘kokonte’ (20p) +groundnut soup | 1.32 | 660 | 0.4 | 871.2 |
| oats +sugar + milk | 0.92 | 283 | 0.4 | 260.08 |
| plain rice + stew | 1.16 | 264 | 0.6 | 306.24 |
| **Fried foods** |  |  |  |  |
| fried chicken | 2.82 | 14 | 0.2 | 39.48 |
| fried chicken sausage | 1.9 | 8 | 0.1 | 15.2 |
| fried egg | 1.57 | 48 | 0.4 | 75.13 |
| fried fish | 4.71 | 11 | 0.2 | 51.81 |
| fried rice | 1.59 | 276 | 1 | 438.84 |
| fried ripe plantain | 2.23 | 32 | 0.1 | 71.36 |
| fried yam | 2.37 | 39 | 0.3 | 92.43 |
| plantain chips | 5.2 | 17 | 0.2 | 88.4 |
| **Fruits** |  |  |  |  |
| banana | 1.06 | 219 | 0.2 | 232.14 |
| water melon | 0.23 | 220 | 0.5 | 50.6 |
| orange | 0.47 | 167 | 0.1 | 78.49 |
| pawpaw | 0.35 | 173 | 0.5 | 60.55 |
| pineapple | 0.49 | 170 | 0.2 | 83.3 |
| **Others** |  |  |  |  |
| boiled egg | 1.56 | 47 | 0.4 | 73.32 |
| soy kebab | 0.94 | 27 | 0.2 | 25.42 |
| popcorn | 5 | 48 | 0.4 | 240 |
| roasted corn | 2.64 | 32 | 0.2 | 84.48 |
| roasted groundnut | 5.88 | 10 | 0.1 | 58.8 |
| roasted groundnut+ maize | 4.26 | 64 | 0.3 | 272.64 |
| Currency conversion at data collection: GHS 1 = 100 p ≈ USD$0.51 – 0.66 | | | | |
