# Supplementary material for: Basic school pupils’ food purchases during mid-morning break in urban Ghanaian schools
Source: PLoS One. 2020 Sep 1;15(9):e0238308. doi: 10.1371/journal.pone.0238308 (PMC7462272; doi:10.1371/journal.pone.0238308)
Supplement: Attachment — Submitted filename: PLOS PONE-D-19-35805.docx [file pone.0238308.s004.docx]

**Manuscript title:** Basic pupils’ food purchases during mid-morning break in urban Ghanaian schools

**Manuscript number:** PONE-D-19-35805

**Reviewer comments**

Dear editor,

Thank you for the opportunity to review this manuscript. Below are my comments

**General comments**

The manuscript is timely for lower middle income settings giving the upsurge in obesity among children and their associated health implications. Since children spend majority of the day when school is in session, they also make decisions regarding what they should eat etc. This decision is usually influence by various factors including meals that are made available within the school and a certain parameter area around the school, what other friends are eating and how much money is giving to the child for snack and food.

My general comment is that this work will benefit from important context information about schools in Ghana, its organization and in particular the operations of school canteens, vendors within and outside of the school compound.

**Specific comments**

**Abstract**

1. Background: The first sentence could be modified to: Unhealthy food vending can expose children to malnutrition and other diet related challenges such as obesity. The study sought to…
2. Again to me unhealthy vending resonate more to the vending environment, hygiene practices etc. than the food and its content (energy density) which the study sought to explore.
3. In the methods section of the abstract, you need to indicate how the retail food environment score was estimated. The results on the score had no connection to the content in the methods section

**Manuscript**

1. Introduction:
   a. The authors should include a special section on Ghana, particularly the basic school environment for public and private schools. This will give useful background for the classification of schools under the methods section.
   b. Page 5: pages 115-116. Usually the structure of an introduction requires that by the time one is at the conclusion, the issues will be in relation to the study setting (in this case Ghana). Presenting a sentence with a New Zealand example, takes the argument out of place. I suggest New Zealand is not mentioned or that sentence is structured in a way that it relates to Ghana.
2. Methods
   a. This section was clearly described. However it is important to explain what table top vendors mean. This may not be a generic/global terminology (page 6, line 144).
3. Results
   a. The age range of pupils-I think 21 is an outlier. If this was used in the calculation of the mean age, it may fail to represent the true mean age. If 21 appeared only once, then I suggest the authors can remove that from the analysis. Unless there were many pupils who were around 21 years.
4. Discussion
   a. This discussion section has been well-written. I only find some of the comparisons with other studies may not be relevant. For example Page 15, lines 336-337, the North America example can be presented differently

Thank you
