# Supplementary material for: Basic school pupils’ food purchases during mid-morning break in urban Ghanaian schools
Source: PLoS One. 2020 Sep 1;15(9):e0238308. doi: 10.1371/journal.pone.0238308 (PMC7462272; doi:10.1371/journal.pone.0238308)
Supplement: Attachment — Submitted filename: Rsponse to reviewer comments(b).docx [file pone.0238308.s005.docx]

Response to Reviewers

Journal Requirements:

When submitting your revision, we need you to address these additional requirements.

1. Please ensure that your manuscript meets PLOS ONE's style requirements, including those for file naming. The PLOS ONE style templates can be found at https://journals.plos.org/plosone/s/file?id=wjVg/PLOSOne_formatting_sample_main_body.pdf and https://journals.plos.org/plosone/s/file?id=ba62/PLOSOne_formatting_sample_title_authors_affiliations.pdf

**Response: The manuscript has been revised to meet PLOS ONE’s style requirements**

2. We note that you have stated that you will provide repository information for your data at acceptance. Should your manuscript be accepted for publication, we will hold it until you provide the relevant accession numbers or DOIs necessary to access your data. If you wish to make changes to your Data Availability statement, please describe these changes in your cover letter and we will update your Data Availability statement to reflect the information you provide.

**Response: The data used in this manuscript has been included with submission**

3. Please include additional information regarding the survey or questionnaire used in the study and ensure that you have provided sufficient details that others could replicate the analyses. For instance, if you developed a questionnaire as part of this study and it is not under a copyright more restrictive than CC-BY, please include a copy, in both the original language and English, as Supporting Information.

**Response: The questionnaire used in this manuscript has been included with submission**

4. Please include captions for your Supporting Information files at the end of your manuscript, and update any in-text citations to match accordingly. Please see our Supporting Information guidelines for more information: http://journals.plos.org/plosone/s/supporting-information.

**Response: Captions for supporting information have been included in the revised manuscript**

REVIEWER 1

| COMMENTS | RESPONSE |
| --- | --- |
| This paper examines food purchases within and outside a sample (n=5) of primary and junior secondary schools in Ghana. It shows that children have easy access to energy-dense, high fat and sugar foods, which may contribute to the nutritional transition in this population. Overall, the paper is written well and it adds to a literature that is limited. However, I feel that it could be strengthened in a number of ways before being published. | Thank you very much for the time spent to make suggestions for improvement. The authors have fully addressed the issues raised to further strengthen the paper. |
| 1. First, the paper documents what children bought, and by type of vendor within or outside school, but it does not really dig deeper in understanding why children made such choices. In the discussions, it mentions convenience and prices as an important driver of food choice, but there could be others such as taste, emulation of peers, status, etc. Do the authors have collected data on those drivers of food choices among this population? It could be really interesting to understand that more. If they have also qualitative data from focus groups to triangulate with the quantitative findings, the paper could be definitely strengthened, especially for formulating more effective policies on food environments and on nutritional choices in Ghana. | The authors acknowledge the depth of understanding that an examination of pupils’ food choices could generate. Unfortunately, the current study did not collect data on the drivers of food choice and is unable to provide this depth of knowledge. We will definitely consider collecting qualitative data in our future studies. |
| 2. The paper would be strengthened by being more specific on its exact contribution to the literature. Especially in the discussions, it mentions many studies from high-income countries, but these have very different food environments and socio-economic drivers of food choices from Ghana. It would be more interesting to compare findings with the literature from low- and middle-income countries, and Sub-Saharan Africa especially. | Thank you. We have included in the current version, the manuscript’s specific contribution to literature. Please see page 17 lines 367 to 373.  In response to your very relevant suggestions, the discussion has been revised; we have excluded comparison of study findings from developed country settings, that we consider not pertinent. Additional literature from Ghana has been used. |
| 3. Similarly, in the introduction the authors should mention why we should care about this specific issue, which is prevention of overweight/obesity and related non-communicable disease. At the moment, this point is lacking. | We very much appreciate the opportunity to strengthen this section of the manuscript. The introduction has been updated accordingly. See page 3 lines 72 to 76. |
| 4. The data from the study are relatively old (2013). Based on the speed of the nutrition transition in low- and middle-income countries, we could expect that purchases of unhealthy foods could be even larger now than when these data were collected. Can the authors discuss this point more? | We completely agree with the reviewer that the data are relatively old compared to the speed of the nutrition transition. However, the current study used broad categorization of foods such as “sweetened drinks”, etc., which will still give us useful data in terms of policy. The authors believe that the transition has more likely impacted on the “variety” or “options” rather than the broad types available. This has been included in the discussion on page 17 lines 377-382. |
| 5. Can they clarify if the government schools are part of the Ghana school feeding program? They mention they do not have a school canteen but it is not clear if such program is implemented there. We know that this program could change dietary behaviours of children. Also, many private schools in Ghana also offer school lunches. The authors should explore the aspect of school lunches further and how this relate to food choices among this population, including these considerations in their discussions as well. | Yes, the Government or Public Schools are involved in the school feeding programme (SFP) and the participating schools are beneficiaries. However, the SFP only targets kindergarten and primary year 1 to 6, excluding pupils in junior high school. Data from the larger study (Ogum Alangea, 2014) showed that only 8% of the beneficiaries pupils reported the SFP as their usual source of food when at school.  The privately owned schools involved in the current study offered compulsory lunch to pupils. Hence, the current study reports on purchases made in addition to the compulsory lunches and other break periods within the school day.  These details have been updated in the introduction (Please see page 5 lines 119 to 124) and discussion on page 13 (line 293-295; 301-304). |
| 6. The paper mentions that it cannot examine drivers of food choices among younger children (up to grade 3) as a limitation. Another limitation however is not examining food choices among older adolescents, as those are even more autonomous than the age groups under consideration in terms of their food choices, and may even more exposed to marketing. Also adolescence is an age in which overweight and obesity start to become manifest among children growing up in low- and middle-income countries (see Schott et al 2019, Economics and Human Biology). In terms of policies, the authors focus on food environments, however nutritional education could also hold promise if combined by shifts towards healthier food environments. Could they discuss this point? | The authors acknowledge the detailed representation of our limitations and agree that nutrition education has a role to play in promoting healthier diets. The discussion has been updated with this detail on page 13 line 289 |
| 8. The authors should discuss why they selected the specific study settings. Also, how informative are these results, e.g. compared to other major cities in Ghana? And do they have any data on rural areas they could compare their results to? | The authors selected the specific study settings due to their similarity to most urban settings in Ghana, especially the major cities and the adjoining towns. Our findings are not applicable to rural areas in Ghana. This has been acknowledged as a limitation in the discussion. See page 16 lines 367- 373. |
| Line 146: stores, kiosks, etc… why do they start with capital letters? Line 148: Snack with capital letter | These errors have been rectified. |
| Line 215: variety of food types – “of” missing | “of” has been inserted into the phrase |
| Table 2 caption: something missing at the end (I guess “by within/outside school”) | The table caption has been revised to, “Frequency of pupil food purchases during first break period by source of foods” |

REVIEWER 2

| COMMENTS | RESPONSE |
| --- | --- |
| The manuscript is timely for lower middle income settings giving the upsurge in obesity among children and their associated health implications. Since children spend majority of the day when school is in session, they also make decisions regarding what they should eat etc. This decision is usually influence by various factors including meals that are made available within the school and a certain parameter area around the school, what other friends are eating and how much money is giving to the child for snack and food. | Thank you for your succinct summary of this work. We sincerely appreciate your time spent in reviewing this work for improvement. |
| My general comment is that this work will benefit from important context information about schools in Ghana, its organization and in particular the operations of school canteens, vendors within and outside of the school compound. | The authors acknowledge this need for detail. More information on the Ghanaian context have been provided in the introduction. See lines 109 -133. |
| 1. Background: The first sentence could be modified to: Unhealthy food vending can expose children to malnutrition and other diet related challenges such as obesity. The study sought to… | Thank you. This revision has been made. |
| 2. Again to me unhealthy vending resonate more to the vending environment, hygiene practices etc. than the food and its content (energy density) which the study sought to explore. | Thank you very much for your comment. The authors explored the nutrient content of vended foods to aid policy decision on food vending within the current socio-economic context which is largely informal and poorly regulated. |
| 3. In the methods section of the abstract, you need to indicate how the retail food environment score was estimated. The results on the score had no connection to the content in the methods section | The abstract has been revised for clarity. It now reads, “About 53% of retailers located within the schools sold relatively healthier food options.” |
| 1. Introduction: a. The authors should include a special section on Ghana, particularly the basic school environment for public and private schools. This will give useful background for the classification of schools under the methods section. | More information on the school environment have been provided. See page 5 lines 109 to 127 |
| b. Page 5: pages 115-116. Usually the structure of an introduction requires that by the time one is at the conclusion, the issues will be in relation to the study setting (in this case Ghana). Presenting a sentence with a New Zealand example, takes the argument out of place. I suggest New Zealand is not mentioned or that sentence is structured in a way that it relates to Ghana. | Thank you for your suggestion. The New Zealand example has been eliminated together with other dispensable comparisons with developed country settings. |
| 2. Methods a. This section was clearly described. However it is important to explain what table top vendors mean. This may not be a generic/global terminology (page 6, line 144). | Thank you for the compliment. A description of “table top” vendors has been provided in parenthesis for clarity. See page 7 line 161. |
| 3. Results a. The age range of pupils-I think 21 is an outlier. If this was used in the calculation of the mean age, it may fail to represent the true mean age. If 21 appeared only once, then I suggest the authors can remove that from the analysis. Unless there were many pupils who were around 21 years. | Thank you for your comment. There were 8 pupils who were age 18 and above. Excluding them from the analysis, the current mean age is 12.8±1.8 years instead of the 12.9[1.9] years. This change has been made in line 208. Changes in frequencies have been updated in tables 1&2. |
| 4. Discussion a. This discussion section has been well-written. I only find some of the comparisons with other studies may not be relevant. For example Page 15, lines 336-337, the North America example can be presented differently | Thank you for your comment. The discussion has been revised to exclude the North American example and other high-income information. |
|  |  |
